# Triple artemisinin-containing combination anti-malarial treatments should be implemented now to delay the emergence of resistance: the case against

**DOI:** 10.1186/s12936-019-2976-7

**Published:** 2019-10-03

**Authors:** Sanjeev Krishna

**Affiliations:** 1 Centre for Diagnostics and Antimicrobial Resistance, Institute for Infection & Immunity, St. George’s, University of London, London, UK; 2 St George’s University Hospitals NHS Foundation Trust, London, UK; 3 Institut für Tropenmedizin, Universitätsklinikum Tübingen, Tübingen, Germany; 4 Centre de Recherches Médicales de Lambaréné, Lambaréné, Gabon

## Abstract

Managing multidrug resistant malaria can be problematic if currently available artemisinin-containing anti-malarial combination treatments are not used appropriately. Here, I debate that the best way to manage multidrug resistant malaria is to make best use of existing treatments and to develop new classes of anti-malarial drugs and not to make ‘triple combination therapies’ when there is already resistance to one or more proposed components.

Almost 3 years after suggesting that declining efficacies of ACT may benefit from ‘inclusive debates’ it is gratifying to implement this suggestion [1]. I am grateful for the invitation to present the case against triple artemisinin-containing combination anti-malarial treatments. This case rests on the same principles that are used to shut down any flawed drug development programme where ‘go no-go’ decisions bear in mind the following considerations:

*Primum non nocere*—‘at least do no harm’ in the context of triple ACT suggests in the first instance that the risk of any toxicities from combinations should be kept to a minimum. Adding anti-malarial combinations to each other will inescapably increase the risks of adverse events, such as hepatic toxicity, cardiotoxicity and other toxicities in ways that may not be predictable from the profiles of existing combinations. At best, such risks will be additive when combined. In addition to toxicity, there is also an increased likelihood that triple combinations will be less well tolerated by patients, so that even if theoretical advantages of improved efficacy were to be shown, a less well tolerated triple combination course may not be taken to completion as often as the conventional ACT.

Affordability—triple combinations will be more expensive than conventional ACT. They will have to be formulated differently, costs of assessing their safety and efficacy will have to be included in final product costings and regulatory approvals will also demand resources.

Even to consider triple combinations, there would have to be clear evidence that we are running out of conventional combinations. We can do much more with existing and still effective conventional ACT rather than spending scarce resources on developing new triples [2, 3]. Despite clamour to the contrary, according to the World Health Organization we are not yet running out of effective artemisinin-based combinations. In addressing the issue of ‘artemisinin resistance’ Dr. Pedro Alonso made it clear that some combinations are still effective in the Greater Mekong Subregion (GMS) where resistance to other artemisinin-based combinations has arisen [4]. It is unsurprising that the funders of studies on ACT in GMS may take a contrary position on the whole concept of artemisinin resistance. Ensuing exchanges between the WHO and the Wellcome Trust left some observers confused whilst providing reassurance that we still have effective treatments for malaria, even in GMS [5].

But what about the risk that even those artemisinin-based combinations that are effective will fail eventually? And the argument that there is urgency now to develop triples so that we are ready for that eventuality? To consider this argument fully, we should first look at the concept of ‘artemisinin resistance’, which has been developed and used by researchers, especially in GMS, to advance their arguments for funding and trials, including no doubt to test triple combinations. ‘Artemisinin resistance’ is defined by the phenotype of delayed parasite clearance when treatment is given with monotherapy (in research studies) or in combination. There are many flaws with this definition, probably one of the most important being that this delayed parasite clearance phenotype is not predictive of treatment failure with a particular ACT [3, 6]. We know that if say mefloquine/artesunate fails in a particular geographic region, this is associated with increased copy numbers of *pfmdr1* in parasites that have become resistant to the mefloquine component of this combination. These parasites can also display the delayed parasite clearance phenotype. Parasites with these characteristics nevertheless can remain sensitive to DHA/piperaquine treatments despite being labelled ‘artemisinin resistant’. How can they be classified as artemisinin resistant if the common effective component of ACT is an artemisinin [2]? We, and others [7, 8], made this argument several years ago and were accused of aiding the spread of artemisinin resistance. We defended ourselves at the time and subsequent clinical and molecular studies are consistent with our suggestions. Understanding of partner drug resistance mechanisms (for example, to piperaquine) has advanced in the intervening years, and the limitations of monitoring for *kelch13* mutations parasites with delayed clearances have also become clearer. Thus, *kelch13* mutations associated with delayed parasite clearance are not predictive of treatment failures with ACT, consistent with the lack of such predictive value of the phenotype itself [9].

Neither are those working in GMS arguing for the return of quinine to treat severe malaria in ‘artemisinin resistant’ parasites. The most severely affected patients should be given the most effective drugs to minimise mortality. If ‘artemisinin resistance’ were clinically significant then surely artesunate should no longer be considered the drug of first choice in the region?

So where does that leave usage of ACT? Much more investment and effort should be put into implementation studies for using existing artemisinin-based combinations. After all, it is national malaria control programmes that are responsible for delivering effective therapies. These should be supported by the best available evidence, rapidly obtained, to choose the most effective ACT for a region [3]. Included in this argument is the importance of using drugs that are verified in terms of their composition, so that substandard supplies are not used and then incorrectly claimed to have failed in particular regions [10].

The most effective ACT can be assessed with results from molecular and clinical studies using validated markers for resistance (without reliance on the delayed parasite clearance phenotype). More investment in delivering currently effective artemisinin-based combinations, and conversely in discontinuing ineffective combinations (until they regain effectiveness) will yield immediate improvements in treatment outcomes, and that in a highly cost-effective manner.

If ACT continues to fail in conventional treatment doses, then there is always the option of extending treatment regimens beyond 3 days. This would restore efficacy without adding disproportionately to costs, although monitoring for additional toxicities would still be required [11]. This approach is immediately available and, therefore, the least expensive one compared with developing new drugs or triple combinations.

If triple combinations continue to reassert themselves on drug development agendas, then what principles should guide their development? These principles were summarized decades ago by White and Olliaro, and a couple of points are worth revisiting. Writing about sulfadoxine/pyrimethamine (S/P) combined with mefloquine [12]:

*“This fixed dose combination has never been used extensively, and the benefit of the triple combination over single*-*drug regimens has never been established. …. the combination failed to prevent resistance developing in Thailand where it was deployed from 1984. There were two reasons: (1) P. falciparum was already highly resistant to S/P; and (2) the pharmacokinetics of the drugs were not well matched.”*

Experience with S/P + mefloquine highlights well another key consideration for developing any triple combination, namely that if there is already resistance to one or more components of the combination then there are unlikely to be any lasting benefits from developing triple combinations with them. Most artemisinin partner drugs with reasonably matched elimination half-lives have already selected for resistance in populations where there are treatment failures with ACT (TFACT). After all, there would not be any need to develop triples if conventional ACT was effective, and if an artemisinin-based combination is not active, it should not be included in triples. It would seem to make more sense to use conventional ACT nimbly and responsively in conventional combinations after establishing efficacies.

In the next few years, entirely new classes of anti-malarial will become available through efforts of organizations such as MMV and its collaborators. The greatest priority will be to ensure the best partner drugs for these new classes so that new combination therapies become available. There will undoubtedly be an opportunity cost to the development of new combinations if resources are expended on developing the higher risk triple combination approaches with existing anti-malarials.

We have been hearing for many years about the catastrophe of anti-malarial resistance in GMS, and how resources are needed to contain this phenomenon that puts at risk our global anti-malarial strategy. However, there are only a few hundreds of deaths in GMS from malaria each year, compared with those in sub-Saharan African countries with much higher endemicities [13]. The real failures in controlling malaria stem from escape from vector control, inadequate implementation in some national control programmes and lack of nimbleness in changing treatment regimens in the face of TFACT.

We should stop calling delayed parasite clearance ‘artemisinin resistance’, focus on using existing combinations most effectively and support the development of new classes of anti-malarials. And we should drop the potentially toxic, ineffective and expensive idea of triple combinations along the way.
